# Living with Loss: study protocol for a randomized controlled trial evaluating an internet-based perinatal bereavement program for parents following stillbirth and neonatal death

**DOI:** 10.1186/s13063-022-06363-0

**Published:** 2022-06-06

**Authors:** Siobhan A. Loughnan, Frances M. Boyle, David Ellwood, Sara Crocker, Ann Lancaster, Chrissie Astell, Julie Dean, Dell Horey, Emily Callander, Claire Jackson, Antonia Shand, Vicki Flenady

**Affiliations:** 1 NHMRC Centre of Research Excellence in Stillbirth, Mater Research Institute-University of Queensland, Raymond Terrace, Level 3 Aubigny Place, South Brisbane, QLD Australia; 2 Institute for Social Science Research, The University of Queensland, 80 Meiers Rd, Indooroopilly, QLD Australia; 3 School of Medicine, Griffith University, Parklands Drive, Gold Coast, QLD Australia; 4 Gold Coast University Hospital, 1 Hospital Drive, Southport, QLD Australia; 5 La Trobe University, Plenty Rd &, Kingsbury Dr, Bundoora, VIC Australia; 6 Monash University, 553 St Kilda Road, Melbourne, VIC Australia; 7 The University of Queensland, St Lucia, QLD Australia; 8 Faculty of Medicine and Health, The University of Sydney, Camperdown, NSW Australia

**Keywords:** Stillbirth, Neonatal death, Pregnancy loss, Perinatal, Internet, Online, Bereavement, Grief, Distress, Anxiety

## Abstract

**Background:**

Stillbirth and neonatal death are devastating pregnancy outcomes with long-lasting psychosocial consequences for parents and families, and wide-ranging economic impacts on health systems and society. It is essential that parents and families have access to appropriate support, yet services are often limited. Internet-based programs may provide another option of psychosocial support for parents following the death of a baby. We aim to evaluate the efficacy and acceptability of a self-guided internet-based perinatal bereavement support program “Living with Loss” (LWL) in reducing psychological distress and improving the wellbeing of parents following stillbirth or neonatal death.

**Methods:**

This trial is a two-arm parallel group randomized controlled trial comparing the intervention arm (LWL) with a care as usual control arm (CAU). We anticipate recruiting 150 women and men across Australia who have experienced a stillbirth or neonatal death in the past 2 years. Participants randomized to the LWL group will receive the six-module internet-based program over 8 weeks including automated email notifications and reminders. Baseline, post-intervention, and 3-month follow-up assessments will be conducted to assess primary and secondary outcomes for both arms. The primary outcome will be the change in Kessler Psychological Distress Scale (K10) scores from baseline to 3-month follow-up. Secondary outcomes include perinatal grief, anxiety, depression, quality of life, program satisfaction and acceptability, and cost-effectiveness. Analysis will use intention-to-treat linear mixed models to examine psychological distress symptom scores at 3-month follow-up. Subgroup analyses by severity of symptoms at baseline will be undertaken.

**Discussion:**

The LWL program aims to provide an evidence-based accessible and flexible support option for bereaved parents following stillbirth or neonatal death. This may be particularly useful for parents and healthcare professionals residing in rural regions where services and supports are limited. This RCT seeks to provide evidence of the efficacy, acceptability, and cost-effectiveness of the LWL program and contribute to our understanding of the role digital services may play in addressing the gap in the availability of specific bereavement support resources for parents following the death of a baby, particularly for men.

**Trial registration:**

Australian New Zealand Clinical Trials Registry, ACTRN12621000631808. Registered prospectively on 27 May 2021.

**Supplementary Information:**

The online version contains supplementary material available at 10.1186/s13063-022-06363-0.

## Administrative information

**Table:** 

Title	Living with Loss: Study protocol for a randomised controlled trial evaluating an internet-based perinatal bereavement program for parents following stillbirth and neonatal death
Trial registration	Australian New Zealand Clinical Trials Registry (ANZCTR), ACTRN12621000631808, registered prospectively on 27/05/2021 https://www.anzctr.org.au/Trial/Registration/TrialReview.aspx?id=381231&isReview=true. ANZCTR collects and publicly displays the WHO Trial Registration Data Set.
Protocol version	14^th^ March 2022; version 1.1
Funding	The Living with Loss project is supported by the Australian Government’s Medical Research Future Fund (MRFF) Rapid Applied Research Translation program grant awarded to Health Translation Queensland (formerly Brisbane Diamantina Health Partners; BDHP).
Author affiliations	^1^NHMRC Centre of Research Excellence in Stillbirth, Mater Research Institute-University of Queensland, Raymond Terrace, Level 3 Aubigny Place, South Brisbane QLD AUSTRALIA ^2^Institute for Social Science Research, The University of Queensland, 80 Meiers Rd, Indooroopilly QLD AUSTRALIA ^3^School of Medicine, Griffith University, Parklands Drive; ^4^Gold Coast University Hospital, 1 Hospital Drive, Southport QLD AUSTRALIA ^5^La Trobe University, Plenty Rd &, Kingsbury Dr, Bundoora VIC AUSTRALIA ^6^Monash University, 553 St Kilda Road, Melbourne VIC AUSTRALIA ^7^The University of Queensland, St Lucia QLD AUSTRALIA ^8^Faculty of Medicine and Health, The University of Sydney, Camperdown NSW AUSTRALIA
Name and contact information for the trial sponsor	Funding is administered by The University of Queensland for Health Translation Queensland (formerly Brisbane Diamantina Health Partners) and sub-contracted to the Centre of Research Excellence in Stillbirth (Stillbirth CRE), Mater Research Institute-University of Queensland. Research office located at: Cumbrae Stewart building 72, St Lucia campus, Brisbane Queensland Australia; Phone: +61 7 3365 3560
Role of sponsor	The sponsor has no role in the design of this study and will not have any role during its execution, analyses, interpretation of data, or decision to submit results.
Composition, roles and responsibilities	**Coordinating centre and data management team:** The Centre of Research Excellence in Stillbirth (Stillbirth CRE), Mater Research Institute-University of Queensland based in Brisbane Queensland Australia is responsible for the overarching conduct and coordination of this study. The coordinating research team (SL, SC, AL, CA, FB, DE, VF) are based at the Stillbirth CRE and are responsible for the day-to-day conduct of the study including participant monitoring and data management. **Steering committee:** An advisory group has been established to oversee and review conduct of this study with meetings scheduled every three months, and monthly updates provided via email correspondence. This steering group includes bereaved parents, health care professionals, clinicians, researchers, and parent support and advocacy organisations. The role of this group is to provide specialist knowledge of perinatal bereavement care and mental health, lived experience of perinatal loss, and trial support and advice throughout all stages of the study, including development, evaluation, and dissemination of findings.

## Background

The death of a baby during pregnancy or soon after birth is a devastating outcome for families and remains a public health priority in Australia and internationally. In Australia, eight stillbirths (defined as the birth of a baby without signs of life after 20 weeks gestation) and neonatal deaths (defined as a baby that dies after birth within 28 days) occur each day, with little change in rates over the past 30 years [1–4]. The long-lasting psychological and emotional consequences for parents, families, and care providers, and the wide-ranging impacts on health systems and society are now well-recognized [5, 6]. Bereaved parents report intense sadness, anxiety, guilt, anger, and experiences of stigma, shame, and disenfranchisement of their grief [7, 8]. Perinatal bereavement is also associated with increased risk for persistent psychological difficulties including posttraumatic stress disorder (PTSD), major depressive disorder (MDD), and prolonged grief disorder (PGD) [6, 9].

The provision of respectful and supportive perinatal bereavement care to parents and families around stillbirth and neonatal death is a major contributor to their immediate and long-term wellbeing [10]. Community-based settings are vital to providing ongoing support to parents following hospital discharge, but support received is often inadequate [4, 11, 12]. The Lancet Ending Preventable Stillbirth Series highlighted the unmet needs of bereaved parents following hospital discharge with 31% of women describing their post-hospital care after stillbirth as poor [4, 11]. Critical gaps exist in the transition from hospital-to-home and in providing ongoing bereavement support for families following a baby’s death [4, 11, 12]. Health professionals report barriers to the effective provision of perinatal bereavement care, including insufficient training and experience in bereavement care, a lack of clear care pathways, and limited availability of support systems and structures for parents [5].

Access to a range of bereavement support options is often recommended to meet the varying needs of individuals [13]. Yet little is known about the acceptability and effectiveness of different types of bereavement support, and whether this differs between parents [14]. For men, in particular, the psychological and emotional impact of stillbirth and neonatal death has been under-explored in comparison to women [15–17]. A systematic review by Obst and colleagues highlighted the importance of tailored perinatal bereavement support resources that validated men’s experiences of grief following a baby’s death [18].

Over the past two decades, the digital delivery of mental health services via the Internet has been increasingly used in Australia to reduce barriers to care provision and increase availability and accessibility of services. Internet-based interventions, predominately based on cognitive behavioral therapy (referred to as “iCBT”), are now well-established for the treatment of a range of mental health disorders including anxiety and depression in children and adolescent [19], and general adult [20], and perinatal populations [21–23]. Digital mental health tools and services already form part of routine care in many countries, often operating within a stepped-care approach as a stand-alone service (i.e., low-intensity support) or in conjunction with face-to-face care services (i.e., high-intensity support) [24, 25]. Low-intensity internet-based programs, with or without therapist guidance or coaching, can provide users with a self-managed flexible support option that can be accessible at any convenient time or place (e.g., at home) and can allow for a greater degree of anonymity and privacy [20, 25]. This is possibly important in the context of perinatal bereavement given the death of a baby during pregnancy often receives little community acknowledgement. Many parents may be reluctant to seek help and disclose emotional difficulties to others following a loss that is often misunderstood and subject to social stigma [26]. Telehealth is now in widespread use in maternal healthcare, and many parents would already be likely to have encountered some form of digital service delivery during their care, this potentially adding to the acceptability and familiarity of parents with a digital mode of bereavement support [27].

Internet-based programs for people experiencing bereavement appear promising yet evidence is limited. A systematic review and meta-analysis of internet-based interventions for grief after bereavement [28] found seven studies which demonstrated moderate effects for symptoms of grief, depression, and posttraumatic stress symptoms, and high user satisfaction and intervention quality. To our knowledge, only one RCT has evaluated an internet-based intervention for parents after pregnancy loss. Kersting and colleagues evaluated a 5-week therapist-guided program (delivered in German language) for parents who had experienced miscarriage, termination of pregnancy for medical reasons, or stillbirth [29]. The program was based on CBT and focused on exposure (self-confrontation), cognitive restructuring, and social sharing and included structured personalized writing assignments that were reviewed by an internet-based therapist. Parents (92% were female, mean age of 34 years) that completed the intervention (*n* = 115) demonstrated significantly reduced symptoms of grief, posttraumatic stress, and depression at post-intervention compared with the wait-list control group (*n* = 113). In addition to low attrition rates (14%), significant improvements in symptoms were maintained at 3-month and 12-month follow-ups.

Generally, internet-based interventions for bereavement appear to be most effective for people experiencing significant loss-related distress and complicated grief responses; users that are self-referred; and when the intervention includes some elements of CBT [30, 31]. Further research is needed to determine whether digital support options, including internet-based interventions, can address the gap in the availability of specific bereavement support for parents following the death of a baby, especially men. Future research should also explore the impact of internet-based interventions on perceived psychological or personal growth outcomes after perinatal bereavement, such as posttraumatic growth, resilience, and coping [32–34]. Evaluation of digital support services is particularly important given the COVID-19 pandemic has resulted in many healthcare services and support organizations rapidly pivoting from face-to-face delivery to digital services.

The *NHMRC Centre of Research Excellence in Stillbirth* (Stillbirth CRE) have led the development of a parent-centered internet-based program to improve the emotional wellbeing of parents following stillbirth or neonatal death and expand the availability and accessibility of evidence-based bereavement care options in Australia for parents and healthcare professionals. The aim of this trial is to conduct a RCT to evaluate the efficacy and acceptability of the internet-based Living with Loss (LWL) perinatal bereavement program, compared to standard usual care. We hypothesize that the LWL program will:

- Reduce symptoms of psychological distress compared with usual care.
- Reduce grief intensity, anxiety, depression, decisional regret, and improve quality of life outcomes compared to usual care.
- Be perceived as helpful, with participants reporting high levels of program satisfaction including usability, credibility and quality; and low attrition rates.
- Be cost-effective compared with usual care.

## Methods/design

### Design and setting

The LWL trial is a two-arm, parallel group, superiority trial comparing the intervention arm (LWL group) with a care as usual control arm (CAU group). This trial will be conducted and reported according to the CONSORT-EHEALTH [35] and Standard Protocol Items: Recommendations for Interventional Trials (SPIRIT) 2013 statement ([36]; see Additional file 1). This 21-week trial includes three assessment timepoints: (1) baseline; (2) post-intervention; and (3) 3-month follow-up. This trial has been approved by the Mater Misericordiae Ltd Human Research Ethics Committee (HREC/MML/70343) and registered with the Australian New Zealand Clinical Trials Registry (ACTRN12621000631808). This trial is being conducted by the Stillbirth CRE, Mater Research Institute-University of Queensland (MRI-UQ) in Brisbane, Queensland Australia. The Stillbirth CRE leads a national program of research and implementation to reduce the number of preventable stillbirths and neonatal deaths in Australia and internationally, and to improve care for women, families, and the community when the death of a baby does occur. This trial is being conducted online with no face-to-face contact with participants. All trial procedures and outcome assessments are completed online via the LWL course delivery system.

### Development of an internet-based perinatal bereavement program

The LWL program focuses on the psychosocial needs of parents and aims to provide a range of practical strategies in self-care and emotional management. Program content was developed by a team of clinicians, researchers, parent support and advocacy organizations, and bereaved parents who collectively provided specialist knowledge of perinatal bereavement care and mental health, and lived experience of perinatal loss. Development was guided by the principles of co-design with emphasis placed on engagement with end-users and the views of lay people [37]. Qualitative semi-structured interviews were conducted with parents and healthcare professionals in community settings. Interviews explored barriers and enablers to bereavement care support options, and the needs and preferences of parents and healthcare professionals for an internet-based perinatal bereavement program.

The LWL program consists of six modules covering a broad range of topics that bereaved parents and healthcare professionals have highlighted as important (see Table 1) [11, 38]. In the absence of a Core Outcome Set (COS) for stillbirth and neonatal death, program topics were mapped to the COS identified for coping and wellbeing in bereavement for adults in palliative care settings (see Table 1) [13]. Program content is based on a range of cognitive and behavioral approaches to bereavement including strategies from cognitive behavioral therapy (CBT), acceptance and commitment therapy (ACT), mindfulness, and compassion-focused therapy. In line with the current evidence base for perinatal bereavement, program content focused on normalizing and validating the individual grief experience and coping processes and avoided pathologizing grief. Content was written from the perspective of the dual-process model of coping with bereavement which suggests individuals oscillate between loss-oriented and restoration-oriented coping and highlights the various cognitive, behavioral, emotional, relational, and motivational impacts of bereavement [39].

**Table 1:** Components of the *Living with Loss* online perinatal bereavement program

Module	Topic	Outcome^**a**^
**Module 1: Understanding grief**	• Understanding grief experience • Managing and coping with behavioral and physical grief reactions (e.g., tension) • Understanding the difference between grief and anxiety, depression, and trauma; including referral pathways for support • Activity planning including self-care and sleep quality • Mindfulness exercise and targeted relaxation and stress reduction techniques	Acceptance of grief as normal; increased understanding of the experiences of challenging mental and emotional states (e.g., feelings of tension, panic, distress or worry); improved daily functioning and increased participation in regular activities.
**Module 2: Managing Intense Feelings**	• Understanding the emotional impact of grief and loss • Identifying and accepting intense feelings such as anger, guilt, blame, regret; moving from self-criticism to self-compassion • Exploring creative practices such as self-compassion through writing • Mindfulness exercise including elements of acceptance and commitment therapy (ACT)	Improved coping with negative and challenging emotional states and overwhelming feelings of grief.
**Module 3: Balancing Thoughts**	• Understanding the cognitive impact of grief and loss; how thoughts, feelings, and actions interact • Identifying and reframing unhelpful thoughts through a thought monitoring strategy • Mindfulness meditation and grounding activity to reduce anxiety	Improved coping with negative and challenging mental states and overwhelming thoughts of grief.
**Module 4: Facing Hard Situations and Conversations**	• Understanding the social and functional context of grief • Identifying and planning for difficult situations and times ahead (e.g., memories, anniversaries) using structured problem-solving and planners (e.g., return to work) • Managing worried and repetitive thoughts through scheduled worry time and self-compassion breaks • Mindfulness meditation and self-compassion-focused exercise	Practical strategies for social and occupational reintegration; increased participation in work and social activities; confidence accessing support when needed.
**Module 5: Strengthening Relationships and Communication**	• Understanding the impact of grief on relationships including differences in grief between partners and family members • Identifying types of support (practical, emotional) including strategies for how to ask and access help and importance of connectedness with others • Strategies to communicate and set helpful boundaries with others • Mindfulness meditation and self-compassion-focused exercise	Effective strategies for enhancing communication and connectedness with others; improved relationships and social functioning.
**Module 6: The Future**	• Understanding how grief can change over time • Exploring ways to find meaning in grief and renewed sense of purpose in life • Planning for the future and accessing appropriate emotional support when needed • Mindfulness meditation and self-compassion-focused exercise	Finding meaning in grief over the longer term; finding balance between grief and life going forwards; sense of meaning and purpose in life.

^a^Mapped to COS for coping and wellbeing in bereavement [13]

The LWL program is delivered via a custom-built online learning management system and viewed by parents on a computer, tablet, or smartphone with an internet connection. To encourage engagement with the program, each module consists of three sections. First, a short series of illustrated parent stories is viewed which delivers content through dialogue between several characters. This targeted style of information delivery has been shown to be acceptable in other digital mental health programs for perinatal women [21, 22]. This approach allows for a range of character experiences and engagement with different bereavement support services (e.g., one-on-one counselling; community-based support group) to be included which aims to normalize and validate varied individual experiences. Summary information is then displayed on-screen over several brief pages to provide parents with more information addressing key topics and strategies. This information is consolidated via 1–2 exercises or activities which can be completed on-screen and then downloaded and/or printed for later reflection and use. Each module concludes with a grounding mindfulness or compassion-focused meditation. Throughout each module, information is provided at several points to link parents with further support options if needed (e.g., peer and mental health telephone support). A library of resources accessible throughout the program also provides parents with additional information (e.g., websites, books, reading material).

Participants are required to complete the introductory module (Module 1). Once this is accessed, all other modules will become available. Apart from this requirement, the program is designed to be flexible so that participants can complete modules in their preferred order and time period. However, participants are encouraged to complete Module 6 last, as this module focuses on planning for the long term and acts as a conclusion to the overall program, although this is not enforced.

### Participants and recruitment

An opportunity sample of self-selecting participants will be recruited via a snowball sampling strategy. Parents and healthcare professionals will be notified of this trial through online and social media advertising, flyers at local maternity services (e.g., Mater Mothers’ Hospital, Brisbane, Australia), and by word of mouth (e.g., healthcare professionals; parent support organizations). All parents interested in participating are directed to the LWL website to review participant information and express interest. This trial aims to recruit at least 75 participants in each study arm (total *N* = 150) which allows for an anticipated dropout rate of 25% (see Statistical methods). Participants will be recruited over a 12-month period.

#### Inclusion criteria

This study will recruit parents who have experienced a stillbirth (defined as a baby that dies before birth and after 20 weeks’ gestation including termination of pregnancy for medical reasons) or neonatal death (defined as a baby that dies after birth and within 28 days) in the past 2 years and more than 8 weeks ago. Additional inclusion criteria include: aged 18 years and older; currently residing in Australia; access to a computer with Internet connection; written and oral fluency in English language; willingness to provide personal contact details (including emergency contact); and willingness to provide informed consent online.

#### Exclusion criteria

The exclusion criteria are as follows: parents in a current pregnancy; those who have experienced an early pregnancy loss (i.e., before 20 weeks’ gestation); stillbirth or neonatal death less than eight weeks ago; or a diagnosis of psychosis, bipolar disorder, or schizophrenia within the last 2 years. Individuals who are experiencing severe symptoms of psychological distress, depression, and/or suicidal ideation at enrolment will also be excluded.

### Procedures

#### Online screening and enrolment

**Fig. 1:**
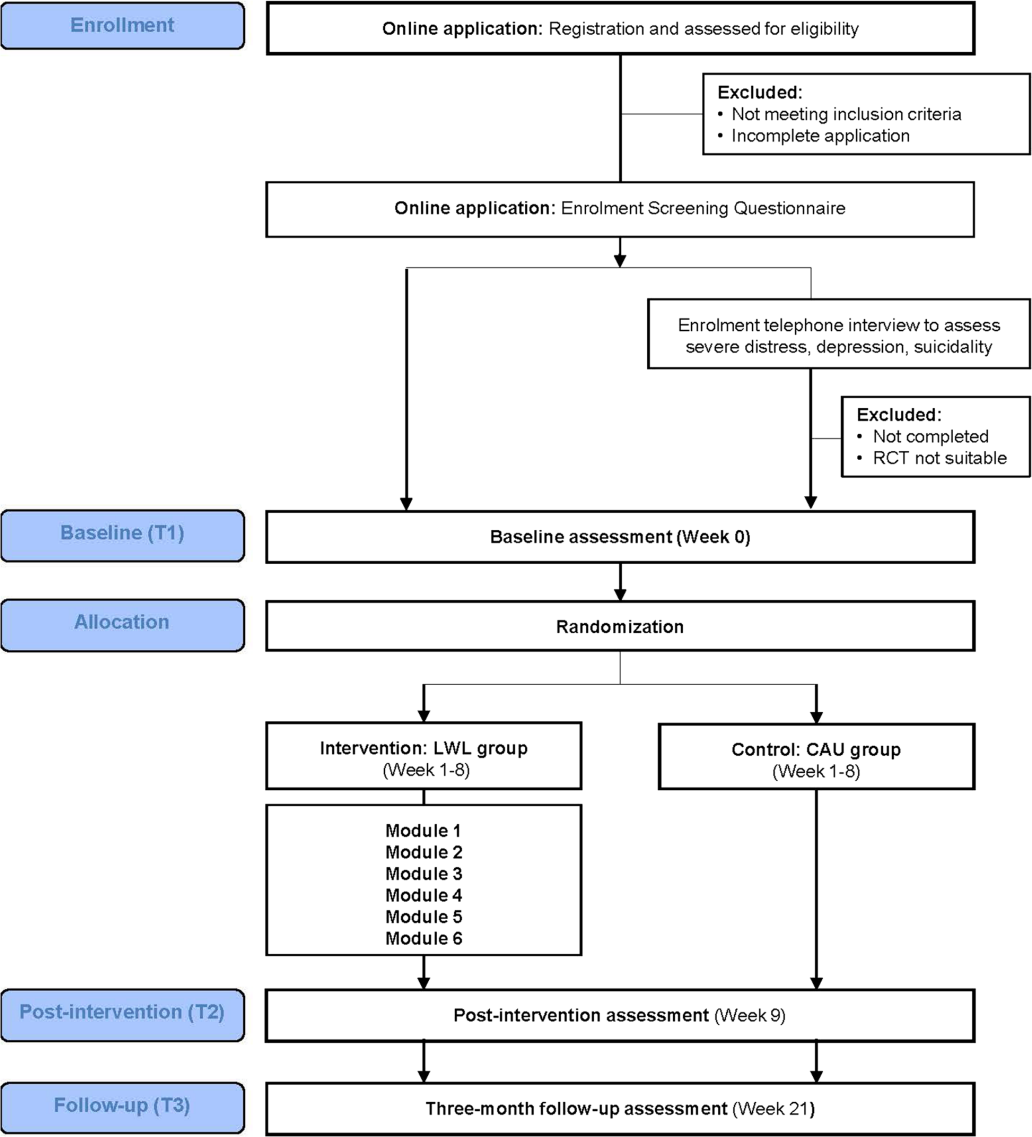
Participant flowchart illustrating enrolment and study procedure

The enrolment and study procedure is outlined in Fig. 1. To enrol, applicants are directed to the LWL website where participant information is provided. To register, applicants are required to read the participant information on-screen, answer eligibility questions based on inclusion and exclusion criteria, and provide online informed consent to participate (by selecting the response option: “Yes, I would like to participate in this research study” on-screen). Applicants that do not meet inclusion criteria are notified on-screen that they are not eligible for this trial and provided with information on parent support organizations. Parents that do not wish to provide consent to participate are asked to close their web browser. Applicants are then asked to complete their personal account details (i.e., personal contact details; emergency contact details; demographics; password for account). All participant account information is confidential and accessible only to the research team based at the coordinating center via the LWL course delivery system.

During enrolment, applicants are also required to complete two baseline questionnaires which act as a screener for severe symptoms of the distress, depression, and/or suicidal ideation exclusion criteria; indicated by a total score of 30 or greater on the Kessler Psychological Distress Scale (K10) [40]; and/or a total score of 14 or greater on the Edinburgh Postnatal Depression Scale (EPDS) [41]; and/or a score of 1, 2, or 3 on Item 10 of the EPDS, respectively. There are two outcomes following this screening questionnaire:

1. Applicants that screen negative (do not meet this exclusion criteria) will be eligible to participate and will proceed to the first assessment timepoint to complete baseline questionnaires prior to randomization.
2. Applicants that screen positive will have their application paused and be notified on-screen and via automated email that the trial clinician will be in contact via telephone to finish their application. During this enrolment interview, the trial clinician will conduct a risk assessment and use clinical judgment to determine eligibility. This is in line with most efficacy studies of digital mental health interventions (i.e., considered low-intensity support) which exclude individuals that are experiencing severe depressive symptoms and are at risk of suicide, and refer to face-to-face services (i.e., high-intensity support [42];. In addition to understanding the applicant’s individual circumstances and support network, this interview will take into consideration that this is an RCT (individuals can be allocated to the control arm) evaluating a self-guided intervention (no therapist support or coaching; content does not address severe depression or suicidal ideation) and delivered solely via the Internet (with no direct participant contact; participants can reside anywhere in Australia). Following the interview, eligible applicants will be asked to return to the LWL website to continue their application. For applicants that are not eligible, referral information for support services will be discussed and provided via email.

#### Randomization and allocation procedures

Following completion of baseline questionnaires (timepoint 1), participants are randomized online between the intervention (LWL group) and care as usual control condition (CAU group). Randomization is based on a 1:1 ratio using random permuted block randomization, with block sizes of 2, 4, and 6 to ensure equal numbers of participants in each study arm. Randomization is stratified by two variables: relationship to baby (mother who carried baby vs partner) and distress severity at baseline (low distress vs moderate-to-high distress) to ensure equal distribution across both study arms. The allocation sequence was generated by a biostatistician and uploaded to the LWL course delivery system by an IT consultant, both independent to the conduct of this trial. The course delivery system is coded to randomize participants, based on the allocation sequence, immediately following completion of baseline questionnaires. The allocation sequence is concealed from the research team for the duration of the study. Once participants have been randomly allocated to a study arm, they will be automatically logged into their online participant portal and notified of group allocation on their home page. Once allocated to a study arm, both participants and research personnel will not be blinded to group allocation.

Participants in both study arms will be able to access usual care and support services during the 21-week trial period. Such care is expected to include a heterogenous mix of professional and non-professional supports and interventions, and will be assessed at each primary timepoint. Contact between participants and research personnel will be: (1) in response to participant request for technical support; (2) initiated by the trial clinician for the purpose of safety monitoring; or (3) initiated by the research team if a participant has been inactive for more than 14 days following assessment timepoints. No further outcome data will be collected for participants that withdraw or drop out. Participants are encouraged to tell their general practitioner (GP; Family Doctor) that they are participating in this research trial as part of the intervention or control study arm.

#### Intervention (LWL group)

Participants allocated to the intervention study arm will be provided immediate access to the LWL program via their participant portal. The active study period for completion of the intervention is 8 weeks, with participants encouraged to login and review a module each week via automated email reminders. As the LWL program is being evaluated as a self-guided low-intensity intervention, no telephone or email therapist support or coaching for program content will be provided (e.g., around use of strategies). Participants will receive email notifications and reminders to login to their participant portal and complete the relevant study questionnaires when available. Once all three assessment timepoints have been completed, participants will have completed their participation in this trial. Participants in the intervention arm (LWL group) will be invited to complete a 6-month follow-up assessment following completion of the LWL program.

#### Control (CAU group)

Participants allocated to the control study arm will be notified on the homepage of their participant portal that they have completed the first stage of the research study (timepoint 1; baseline questionnaires), with two further stages to be completed in 8 weeks’ time (timepoint 2); and 3 months’ time (timepoint 3). Participants will receive email notifications and reminders to login to their participant portal and complete the relevant study questionnaires when available. Once all three assessment timepoints have been completed, participants in the control arm will have completed their participation in this trial and will be provided the option to access the LWL program through their participant portal.

#### Safety monitoring

Two primary strategies are in place to monitor participants and ensure their safety throughout the trial. Participant progress will be monitored by the trial clinician to identify elevated distress or risk of harm. The trial clinician (AL) is a registered psychologist in Australia with clinical experience in managing perinatal mental health difficulties and diagnoses. Both the participant and the trial clinician will receive automated notification emails if severe symptoms of distress (i.e., total score ≥30 on the K10), depression (i.e., total score ≥13 on the EPDS), or thoughts of self-harm (i.e., item 10 on the EPDS, item 8 on the Perinatal Grief Scale-Short Form (PGS-SF) [43], or item 16 on the Assessment of Quality of Life (AQoL-8D) [44] are indicated at any of the primary timepoints. This notification email will encourage participants to seek support from their support network (e.g., partner, family member) and health professional (e.g., GP/Family Doctor), and if feeling unsafe, to contact emergency or crisis support services. Participants will be contacted by the trial clinician within 72 h via telephone to conduct a risk assessment and determine whether the participant is still eligible to continue in the study, and will be provided assistance to find appropriate support and clinical care if required.

### Outcomes

Primary and secondary outcomes will be collected at three primary timepoints: baseline, post-intervention, and 3-month follow-up (see Table 2).

**Table 2:** Schedule of outcome measures and timepoints

	Baseline	Active study period^a^		Post-intervention	3-month follow-up
		Weekly	End of each module		
***Primary outcome***					
K10	x*	x		x	x
***Secondary outcomes***					
PGS	x			x	x
GAD-7	x			x	x
EPDS	x*			x	x
BGQ	x				
RSA	x				
DRS	x			x	x
Support use	x			x	x
AQoL-8D	x			x	x
HSUS	x			x	x
***Acceptability outcomes***^a^					
CEQ			x^b^		
MSS			x		
PSQ				x	

*K10* Kessler Psychological Distress Scale, *PGS* Perinatal Grief Scale, *GAD-7* Generalized Anxiety Disorder Scale, *EPDS* Edinburgh Postnatal Depression Scale, *BGQ* Brief Grief Questionnaire, *RSA* Resilience Scale for Adults, *DRS* Decisional Regret Scale, *CEQ* Credibility and Expectancy Questionnaire, *MS* Module Satisfaction Scale, *PSQ* Program Satisfaction Questionnaire, *AQoL-8D* Assessment of Quality of Life, *HSUS* Health Service Utilization Scale

*Completed during enrolment screening questionnaire; ^a^LWL group only; ^b^end of Module 1 only

#### Primary outcome—psychological distress

The Kessler Psychological Distress 10-item scale (K10) [40]—a self-report scale to assess symptoms over the past 4 weeks. Items are rated on a 5-point scale from 1 (none of the time) to 5 (all of the time). In addition to the three primary timepoints, participants in the LWL group will also complete the K10 once per week while completing the intervention to monitor for potential harmful effects of the intervention such as symptom deterioration [45]. The K10 has strong psychometric properties, is sensitive to change (i.e., in tracking symptom changes in response to treatment [46]), and has been used in efficacy studies of digital mental health interventions including perinatal populations [21, 22] and in the context of grief [47, 48].

#### Secondary outcomes

- *Perinatal Grief Scale – Short Form* (PGS-SF) [45] is a 33-item self-report measure of behavioral and affective symptoms of grief and symptoms specific to perinatal loss. Statements of thoughts and feelings are divided into three subscales: “active grief,” “difficulty coping,” and “despair” and are rated on a 5-point scale from 1 (strongly agree) to 5 (strongly disagree). Higher scores are reflective of more intense grief. The PGS-SF has been widely used and translated into multiple languages [49].
- *Generalized Anxiety Disorder Scale, 7-Item* (GAD-7) [50] is a self-report measure to assess generalized anxiety symptoms over the past 2 weeks and is sensitive to the presence of generalized anxiety disorder, social phobia, panic disorder, and posttraumatic stress disorder [51]. Participants are asked to rate on a 4-point scale from 0 (not at all) to 3 (nearly every day) whether they have been bothered by anxiety symptoms including nervousness, inability to stop worrying, excessive worry, restlessness, difficulty relaxing, irritation, and feeling afraid. Total scores range from 0 to 21, with higher scores indicating more severe anxiety symptoms. A total score of 8 or more indicates the likely presence of an anxiety disorder.
- *Edinburgh Postnatal Depression Scale* (EPDS) [41] is a 10-item self-report screening measure to assess antenatal and postpartum depressive symptoms over the past 7 days. Items are rated on a 4-point scale from 0 (not at all) to 3 (most of the time). Total scores range from 0 to 33, with a score of 12 or greater indicating possible depression. The EPDS has strong psychometric properties, has been validated in women and men [52], and translated and validated in 20 languages [53].
- *Brief Grief Questionnaire* (BGQ) [54] is a 5-item self-report measure for screening complicated grief symptoms, including difficulty accepting the death, grief interference in current life, troubling thoughts related to the death, avoidance of reminders of the loss, and feeling distant from others. Items are rated on a 3-point scale from 0 (not a lot) to 2 (a lot) with a total score of five or more indicating the presence of complicated grief symptomology [55].
- *Decisional Regret Scale* (DRS) [56] is a 5-item self-report measure assessing the extent of distress or remorse after a health care decision. Items are answered on a 5-point scale from 1 (strongly agree) to 5 (strongly disagree). Total scores range from 0 to 100, with higher scores indicating high regret [56].
- *Resilience Scale* (RS-14) [57] is a 14-item self-report measure of personal resilience, defined by the individual’s capacity to cope with stress and thrive despite life challenges. Responses are scored on a 7-point scale from 1 (strongly disagree) to 7 (strongly agree). Scores range from 14 to 70, with higher scores indicating greater resilience. The RS-14 has been validated in bereaved parent populations [34].
- *Use of other supports and/or treatments*: At each primary timepoint, participants will be asked to provide details of other support services they have accessed before and during the trial period including both informal (e.g., family, friends, spiritual advisor) and formal supports (e.g., mental health professional, parent support organizations).

#### Health economic outcomes

##### Quality of life and health service utilization

Quality of life will be assessed according to the Assessment of Quality of Life (AQoL-8D) [44], a 35-item self-report scale to assess health-related quality of life across eight dimensions: independent living, pain, senses, mental health, happiness, coping, relationships, and self-worth. Health service utilization will be assessed using a 23-item self-report questionnaire developed for this trial to explore health service utilization of nine services: general practitioners/nurse practitioners, psychology services, specialist services, hospital emergency department services, hospital inpatient services, hospital outpatient services, medications, other services; and out-of-pocket service fees incurred.

#### Acceptability outcomes—LWL group only

##### Expectancy of benefit

Credibility and expectancy of benefit will be assessed after completion of the first module. Two questions assess expectations around the usefulness and benefit of the LWL program (e.g., “How useful do you think the information or modules of the LWL program will be?”) and one question assesses concerns about the program (e.g., “Do you have any concerns about the program?”) with all responses rated on a 5-point scale from “very useful” to “not useful”. An open-ended question invites any further comments.

##### Engagement with LWL program

Adherence to the program will be reflected in the mean number of sessions completed by participants. Other website analytic tools will allow program usage (e.g., number of times logged in, number of times a module has been accessed and completed, time spent per module, and order modules were completed) to be assessed.

##### Program satisfaction and experiences

Participants are asked to provide feedback on the program: (1) immediately after completing a module and (2) at post-intervention. At the end of each module, participants are asked to rate five statements on a 5-point scale (from strongly agree to strongly disagree) which assess the perceived helpfulness and usefulness of each module’s topics, themes, activities and strategies, and ease of use. Participants are also asked to provide descriptive feedback around aspects of the module that they did not find helpful, aspects which they felt were missing, and to provide suggestions for revisions and improvements. Following completion of the program (and end of active trial period), participants are asked 12 questions around their overall experience which include a range of open-ended, brief rating scales, and multiple-choice responses. Questions assess the perceived helpfulness of program content (e.g., character stories, strategies, activities) and ease of use, feedback on desired involvement of healthcare professionals for additional support (e.g., weekly check-ins, telephone support), and how likely the participant would be to recommend the LWL program to their GP or other bereaved parent. These questionnaires were developed specifically for this trial and are based on similar intervention satisfaction measures (i.e., the *Treatment Satisfaction Questionnaire* (TSQ) [58].

### Data management and monitoring

This study is co-ordinated and managed by the Stillbirth CRE located at Mater Research Institute (MRI) within The University of Queensland Faculty of Medicine in Brisbane, Australia. All participation procedures and data collection will be conducted through the custom-built LWL online course delivery system managed by the coordinating research team based at the Stillbirth CRE. Re-identifiable data only (i.e., coded) will be exported weekly from the LWL course delivery system by a member of the research team and stored in accordance with Stillbirth CRE data management specifications. There is no data monitoring committee or planned interim analyses given this is a short-term small study involving a low-risk intervention. Participant safety is monitored by the trial clinician as part of the intervention. A steering group has been established to oversee and review data validity and overall conduct of the trial. This steering group includes clinicians, researchers, parent support and advocacy organizations, and bereaved parents who collectively provide specialist knowledge of design and conduct of RCTs, perinatal bereavement care and mental health, and lived experience of perinatal loss.

### Statistical methods

#### Sample size calculation

As the primary outcome in this trial is both continuous and measured longitudinally, we employed the sample size calculation suggested by Diggle and colleagues [59]. Assuming a significance level of 0.05, 80% power, a moderately strong within-subject correlation (*ρ* = 0.5), three timepoints, and an anticipated dropout rate of 25%, a minimal total sample size of 150 participants is required to detect a standardized difference (Cohen’s *d*) of 0.5 (i.e., a “moderate” difference between groups). This power calculation was informed by published RCTs of brief digital mental health interventions for posttraumatic stress and prolonged grief in parents after the loss of a child during pregnancy [29] and psychological distress, anxiety, and depression in postpartum women [22].

#### Data analyses

All analyses will be undertaken in Statistical Package for the Social Sciences (SPSS) version 25 (IBM SPSS, IBM Corp., Armonk, NY, USA) using re-identifiable (i.e., coded) data. Data analysts will not be blinded to study arm. Descriptive statistics will be used to summarize participant demographic and obstetric data. Group differences in baseline variables will be examined using cross-tabulations, independent *t*-tests, and regression analyses, with differences in baseline outcome measures controlled for in further analyses. To determine program efficacy, intention-to-treat linear mixed models will be estimated for each outcome measure, with restricted maximum likelihood (REML) estimation used to account for missing data due to participant dropouts [60]. These models will yield more accurate estimates of effect compared to completer as they account for the unbalanced nature of the data [60, 61]. Mixed models will be estimated separately for each outcome variable, with time, group, and time by group interaction entered as fixed factors. Comparisons within and between groups from baseline to 3-month follow-up for each group will be undertaken for outcomes. Between-group effect sizes will be calculated using the pooled standard deviation of the estimated marginal means and adjusted for sample size (Hedges *g*). Effect sizes of 0.20, 0.50, and 0.80 are considered small, moderate, and large respectively [62]. Descriptive statistics will be used to summarize participant engagement, adherence, and program satisfaction data.

A cost-utility analysis comparing the program’s efficacy and acceptability against usual care in a primary care setting will be conducted. Analyses will be based on direct and indirect costs associated with program development, evaluation, and participants’ social opportunity costs (e.g., work productivity, absenteeism, and presenteeism). The health economic evaluation protocol and findings from this trial will be published separately.

## Discussion

The National Stillbirth Action and Implementation Plan (NSAIP) outlines priorities to address stillbirth in Australia including improving the quality and accessibility of bereavement care and support received by parents [63, 64]. This study will provide evidence of whether a specialized internet-based perinatal bereavement program can address the psychosocial needs of bereaved parents. If proven to be beneficial, the LWL program will provide parents in Australia with an evidence-based, easy-to-use, and accessible support option following stillbirth or neonatal death and may also help address the geographical disparity in access to bereavement services and resources. In 2018, approximately 30% of stillbirths and neonatal death occurred in rural and remote regions of Australia [1]. Compared to those in metropolitan areas, parents in rural and remote regions have limited access to face-to-face support services and the specialized bereavement care that is often needed following the death of a baby [12].

While this RCT aims to address gaps in the perinatal bereavement literature, the following limitations are noted. First, we expect both the CAU control condition and LWL group to include a heterogenous mix of professional and non-professional supports throughout the trial period. It is not possible to compare the efficacy and acceptability of this intervention in comparison with other bereavement supports or services as an active control condition was not included. Second, there is no standardized measure of distress for perinatal grief and bereavement, which limits ability to differentiate between clinical levels of distress which may benefit from clinical intervention, and grief symptomatology which does not typically require intervention [65]. It is also important to acknowledge the “digital divide” and inequity of access to digital technology and disparities in technological health literacy for many vulnerable populations, including non-English-speaking communities [66]. In line with other digital mental health research, this study sample may therefore be biased and not representative of all bereaved parents which limits generalizability of findings.

The delivery of timely, accessible, and evidence-based perinatal bereavement care for parents following the death of a baby is a priority in Australia and internationally. Recent research has highlighted the shifting landscape of grief in the digital age and the rapidly growing availability of and engagement with digital resources for support, including forum-based grief websites, virtual groups, and social media sites [67]. This has been particularly evident during the COVID-19 pandemic with face-to-face delivery of grief support either limited or unavailable, and further highlights the importance of rigorous evaluation of online support options.

## Trial status

This trial started recruitment on 28 June 2021. As of 1 February 2022, 62 participants have been enrolled and allocated to a study arm (LWL: *n* = 31; CAU: *n* = 31). Recruitment is ongoing and expected to conclude in June 2022.

## Supplementary Information

**Additional file 1.** SPIRIT 2013 Checklist: Recommended items to address in a clinical trial protocol and related documents*.

## Data Availability

The final trial dataset and corresponding re-identification codebook will be accessed by the trial investigator team only. The funders have no role in the availability of data. The datasets analyzed during the study and statistical code are available from the corresponding author on reasonable request, as is the protocol.

## Abbreviations

AQoL-8D: Assessment of Quality of Life
BGQ: Brief Grief Questionnaire
CBT: Cognitive behavioral therapy
CEQ: Credibility and Expectancy Questionnaire
DRS: Decisional Regret Scale
EPDS: Edinburgh Postnatal Depression Scale
GAD-7: Generalized Anxiety Disorder Scale, 7-item
HSUS: Health Service Utilization Scale
iCBT: Internet-delivered cognitive behavioral therapy
K10: Kessler Psychological Distress Scale, 10-item
LWL: Living with Loss
MSS: Module Satisfaction Scale
NSAIP: National Stillbirth Action and Implementation Plan
PGS-SF: Perinatal Grief Scale, Short Form
PSQ: Program Satisfaction Questionnaire
RCT: Randomized controlled trial
RSA: Resilience Scale for Adults
Stillbirth CRE: NHMRC Centre of Research Excellence in Stillbirth, Brisbane Australia
